# Photoacoustic imaging with an acoustic lens detects prostate cancer cells labeled with PSMA-targeting near-infrared dye-conjugates

**DOI:** 10.1117/1.JBO.21.6.066019

**Published:** 2016-06-30

**Authors:** Vikram Dogra, Bhargava Chinni, Shalini Singh, Hans Schmitthenner, Navalgund Rao, John J. Krolewski, Kent L. Nastiuk

**Affiliations:** a University of Rochester, Department of Image Science, 601 Elmwood Avenue, Rochester, New York 14642, United States; b Roswell Park Cancer Institute, Department of Cancer Genetics, Elm and Carlton Streets, Buffalo, New York 14263, United States; c Rochester Institute of Technology, Carlson Center for Imaging Science, 54 Lomb Memorial Drive, Rochester, New York 14623, United States; d Rochester Institute of Technology, School of Chemistry and Materials Science, 54 Lomb Memorial Drive, Rochester, New York 14623, United States; e Roswell Park Cancer Institute, Center for Personalized Medicine, Elm and Carlton Streets, Buffalo, New York 14263, United States

**Keywords:** photoacoustic imaging, prostate, prostate-specific membrane antigen, near-infrared, aptamer, acoustic lens

## Abstract

There is an urgent need for sensitive and specific tools to accurately image early stage, organ-confined human prostate cancers to facilitate active surveillance and reduce unnecessary treatment. Recently, we developed an acoustic lens that enhances the sensitivity of photoacoustic imaging. Here, we report the use of this device in conjunction with two molecular imaging agents that specifically target the prostate-specific membrane antigen (PSMA) expressed on the tumor cell surface of most prostate cancers. We demonstrate successful imaging of phantoms containing cancer cells labeled with either of two different PSMA-targeting agents, the ribonucleic acid aptamer A10-3.2 and a urea-based peptidomimetic inhibitor, each linked to the near-infrared dye IRDye800CW. By specifically targeting cells with these agents linked to a dye chosen for optimal signal, we are able to discriminate prostate cancer cells that express PSMA.

## 1 Introduction

The clinical management of early stage, organ-confined prostate cancer (PrCa) is challenging. The introduction of serum prostate-specific antigen (PSA) screening in the 1980s led to a spike in the apparent incidence of PrCa, due to the detection of previously underdiagnosed indolent cancers, which grow slowly and do not affect lifespan.^1^ While PSA screening has declined from its peak, most PrCa is still screen-detected and, therefore, likely to be indolent, requiring no treatment. Unfortunately, there is no reliable technology to identify the rare aggressive cancers among the mass of indolent screen-detected PrCa, which has led to frequent overtreatment by surgery or radiation. A lot of effort has been focused on the development of tissue or serum biomarkers to differentiate indolent from aggressive disease, but this has not yet yielded sensitive and specific clinical tools. Consequently, the current clinical paradigm for screen-detected PrCa is active surveillance^2^^,^^3^ (AS): monitoring by serum PSA testing and serial prostate biopsies to determine the grade (Gleason score) and extent of tumor.

There is an urgent need for imaging tools for AS, (i) to confirm the initial PSA-based diagnosis; (ii) to guide biopsies, which are now performed in a blinded manner; and (iii) to monitor tumor volume, which is currently not measureable but may represent a biomarker of progression from indolent to aggressive disease. Multiparametric magnetic resonance imaging (MRI) may fill some of these roles, but it is expensive and technically limited.^4^ In contrast, photoacoustic imaging (PAI), an emerging, noninvasive, functional molecular imaging modality that has not yet entered the clinic, is likely to be less expensive and more portable than any MRI system. The photoacoustic (PA) signal is an ultrasound (US) wave generated by tissue constituents [hemoglobin (Hb), fat, water, and so on] following absorption of short (nanosecond) pulses of laser light in the near-infrared (NIR) spectrum.^5^ PAI can discriminate among such tissue constituents on the basis of optical absorption properties, allowing for PAI spectroscopy, which can detect biological function.^6^

Human PrCa is a viable candidate for PAI since transrectal probes can image the prostate gland *in situ*. Based on US data from human PrCa patients, we estimate that the distance from the rectal wall to the anterior prostate is ∼3.5  cm (VD, BC, JJK, KLN, unpublished data), which is within the range of depth detection of PAI for PrCa.^7^ The most common application of PAI spectroscopy in cancer imaging exploits differences in the absorption spectra of Hb and ${\mathrm{HbO}}_{2}$ and is therefore capable of detecting tumors based on regions of hypoxia, that promote neoangiogenesis and more aggressive cancers.^8^ However, endogenous tissue constituents, such as Hb, generate relatively weak photoacoustic signals (due to a small absorptivity factor or extinction coefficient) and lack cancer specificity. Exogenous agents, such as NIR-absorbing dyes or gold particles, linked to tumor-specific binding molecules, such as antibodies, can act as targeted molecular imaging agents (TMIAs) to facilitate sensitive and specific detection of the corresponding cancer. Several TMIAs-targeting PrCa have been reported, but while overexpressed in some PrCa, the targets (GRPR and Her2) are more widely expressed.^9^ In contrast, PSMA is highly specific and detected on the surface of nearly every human PrCa, with low to moderate expression on noncancer prostate tissue and very low expression outside the prostate, making it an excellent biomarker for molecular imaging of PrCa.^10^ Unfortunately, the FDA-approved application of PSMA detection (ProstaScint) is of limited value because while PSMA is an excellent target, ProstaScint employs a monoclonal antibody against the internal (cytoplasmic) domain of PSMA, and so detects only necrotic cells.^11^ Subsequently, improved PSMA-binding agents have been developed, including a nuclease-stable ribonucleic acid aptamer (A10-3.2) that binds very efficiently.^10^ PSMA also has an unusual extracellular active site encoding glutamate carboxypeptidase activity, allowing for the synthesis of a urea-based peptidomimetic inhibitor (DCL), that has been linked to a NIR dye for successful *in vivo* imaging of PSMA+ mouse xenografts^12^ and for radiometric imaging of PrCa in patients.^13^

## 2 Materials and Methods

**Fig. 1:**
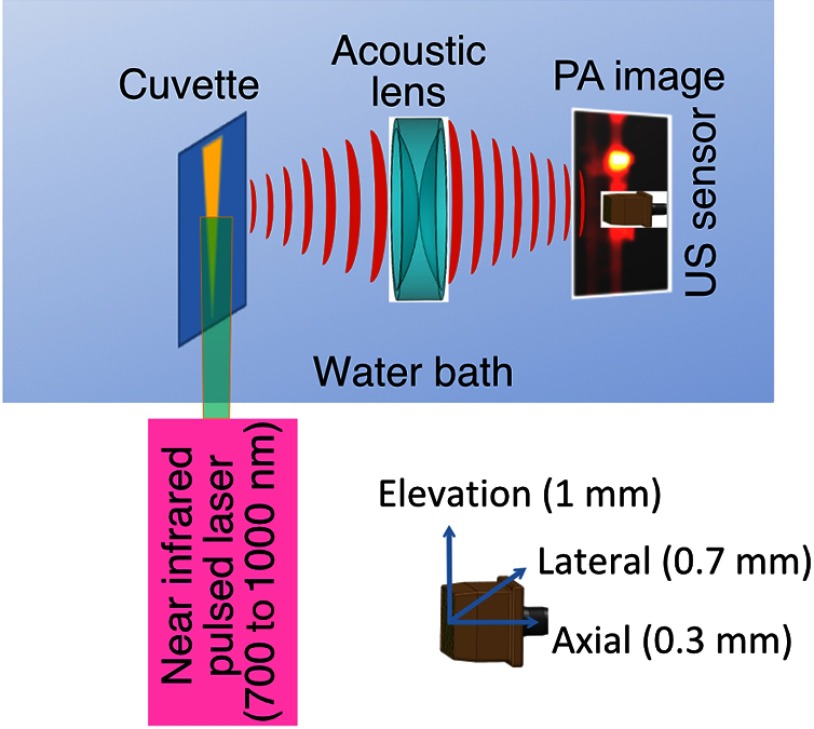
PAI device configuration. A schematic of the sample cuvette in the instrument and the US 32 sensor linear array-transducer elevation stepped to yield the c-scan image (with oriented resolution).

Figure 1 shows the PAI instrument we employed in this study, similar to the prototype we described previously.^14^ Following laser excitation, PA signals from all the absorbers in a small volume of tissue are simultaneously focused on an US detector using an acoustic lens, which corrects for loss of lateral image resolution.^15^ The acoustic lens eliminates the need for expensive and time-consuming off-line computer algorithm-based image reconstruction, reducing errors in the final image. This may facilitate more rapid translation to the clinic. Our PA imaging device is comprised of four modules: (i) a fiber-coupled tunable NIR-pulsed laser with wavelengths ranging from 700 to 1000 nm, pulse repetition frequency of 10 Hz, and pulse duration of 5 ns with a surface laser energy intensity of $∼20\;\mathrm{mJ}/{\mathrm{cm}}^{2}$; (ii) an unfocused 32-element (1×0.7 and pitch of 0.7 mm) linear US sensor array with a central frequency of 5 MHz (range 2 to 8 MHz) and 60% bandwidth; (iii) a spherical acoustic lens with a diameter of 25.4 mm and focal length of 39.8 mm to focus PA signal on the sensors; and (iv) a custom designed 32-channel simultaneous data acquisition unit to amplify (40 to 70 decibels variable gain), digitize (12-bit, 30 MHz), average (8×), and store the received PA signals. To acquire C-scan PA planar images, the device is raster-scanned over the cone shaped (20  mm×1 to 2 mm) sample cuvette using dual-axis stepper motors, while the laser light is delivered using a trans-illumination setup. Previously we used this system to image phantoms^14^ and *ex vivo* human PrCa specimens.^16^

## 3 Results

### 3.1 Identification of Optimal Chromophore for Imaging Agent

**Fig. 2:**
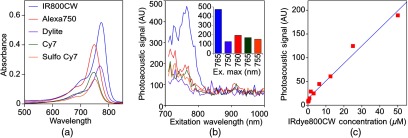
Optical and photoacoustic spectra of candidate NIR dyes. (a) Optical absorption spectra of indicated NIR dyes. (b) Photoacoustic spectra of the five NIR dyes. Inset: Wavelength (5 nm step indicated below) with the maximal photoacoustic intensity (y-axis, AU: arbitrary units) for the NIR dyes [same color coding for dyes in both graph and inset as in (a)]. (c) Photoacoustic signal intensity at 765 nm correlates with IRdye800CW concentration.

In order to improve depth penetration and image quality, exogenous chromophores can be employed as contrast agents as part of a TMIA. Using a laser tuned to the maximum excitation wavelength of the TMIA-chromophore, tumor detection can be greatly enhanced as these exogenous chromophores have absorptivity factors two- to three-orders of magnitude greater than those of endogenous agents such as Hb.^15^ For greatest tissue depth penetration and sensitivity, TMIAs labeled with chromophores that absorb in the “biological NIR window” between 750 and 900 nm circumvent the natural absorbance of Hb, ${\mathrm{HbO}}_{2}$, and $H_{2}O$. To identify a suitable NIR dye, optical absorbance of a 100-μM solution of five commercially available dyes IRDye800CW (Licor), Cy7 (synthesized by H.S.), AlexaFluor750, Cyanine7-sulfo, and Dylight800 (ThermoFisher) was first measured to ensure concordance with supplier data after dilution [Fig. 2(a)]. As expected from the reported peak intensities (lambda max), the Alexafluor 750 and Cy7 have a peak optical absorption when irradiated at 750 nm, Cy7-sulfo at 755 nm, Dylight800 at 785 nm, and the dye IR800CW at 775 nm. The spectra of the photoacoustic signal of a 100-μM solution of each contrast agent was then determined over the 710- to 1000-nm wavelength range [Fig. 2(b)]. The inset shows the maxima of each PA signal and the corresponding wavelength (indicated beneath) in the 730 to 875 nm window (signal below 730 nm reflects laser output fluctuation). Among the five contrast agents/dyes we investigated, IRDye800CW was chosen because it produced the highest PA intensity relative to the other agents and because the peak absorption is well separated from endogenous tissue constituents [Fig. 2(b)]. IRdye800CW was serially diluted in DMSO and water. The resultant PA signal intensity above diluent signal correlates well with concentration [$r^{2}=0.979$; Fig. 2(c)]. Using our PAI apparatus with the acoustic lens in-line, 0.8-μM IRDye800CW is the threshold for detecting signal above noise.

### 3.2 Photoacoustic Signal from Target Expressing Prostate Cells

**Fig. 3:**
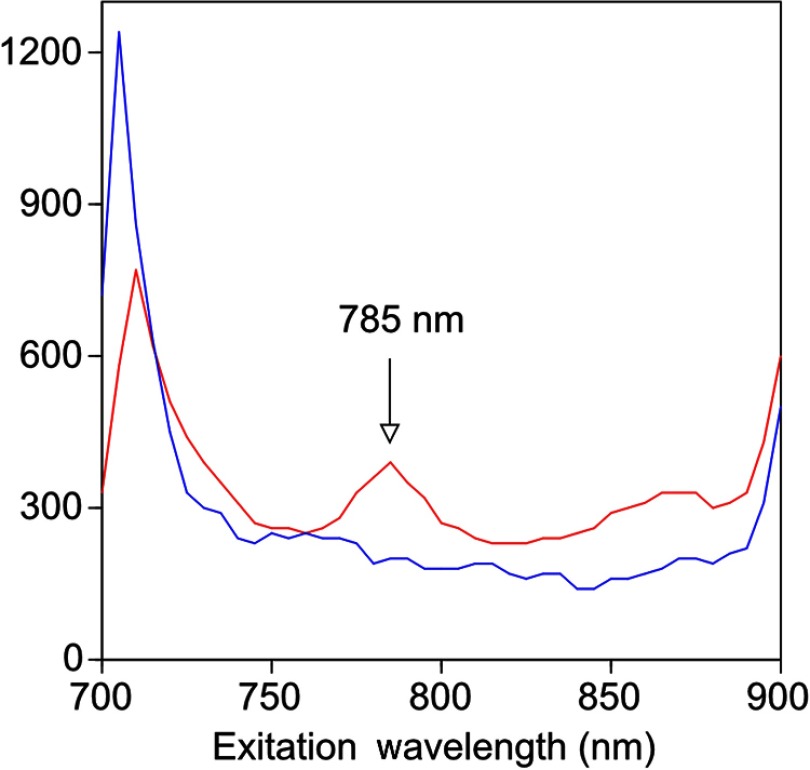
Specific labeling by TMIA for PAI. PSMA+ C4-2 (red) and PSMA- PC3 (blue) prostate cancer cells were labeled with A10-3.3-IRDye800CW and scanned from 700 to 900 nm wavelength laser light in the instrument shown in Fig. 1.

A TMIA was synthesized by labeling the PSMA-specific 10.3.2 aptamer with IRDye800CW (GeneLink). Cells expressing (C4-2) or lacking (PC3) PSMA were incubated in a 4-μM solution of the TMIA in PBS, washed thrice in PBS by centrifugation and resuspension, and examined in our PAI system. The spectra of the photoacoustic signal from C4-2 cells and PC3 cells shows a maximal PA signal difference at 785 nm (Fig. 3). The acoustic lens allows signal detector signal focus from a large incident angle thereby enhancing detection up to fourfold [data not shown (DNS) and Ref. 14]. The 20-nm difference in the wavelength producing peak PA signal between the IRdye800CW alone, at 765 nm, and this 785 nm peak suggests that conjugation of the IRDye800CW to the aptamer to constitute the TMIA, and/or binding of the TMIA to the PSMA molecules on the prostate cell surface, affected the wavelength of absorbance producing the peak PA signal (Fig. 3).

### 3.3 Quantitative Assessment of Aptamer as Targeting Agent

**Fig. 4:**
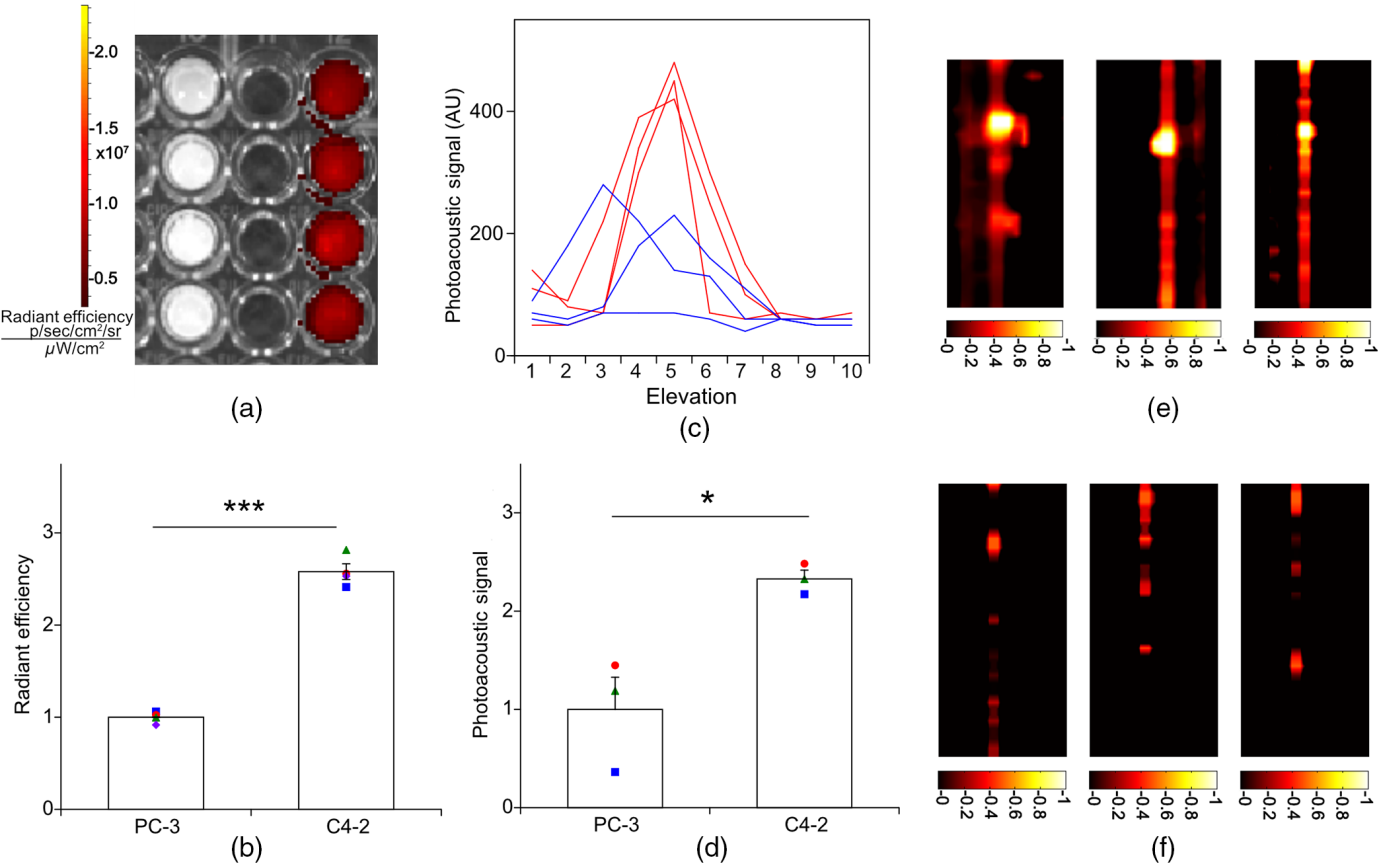
Enhanced photoacoustic signal from PSMA+ C4-2 PrCa cells labeled with A10-3.2-IRdye800CW aptamer-dye. Optical imaging of a multiwell plate containing aliquots of PSMA+ C4-2 (right side four wells) and PSMA- PC3 (left side four wells) cells labeled with aptamer-dye (buffer in middle wells). Fluorescent signal intensity was captured via (a) IVIS and (b) relative intensity plotted, p<0.001. (c) Cells from three of each of the wells in (a) were sequentially loaded into the PAI instrument cuvette (Fig. 1) and PA signal (B-scan) was captured at 785 nm from c4-2 (red lines) and PC3 (blue lines), by scanning the elevation (long) axis of the cuvette, elevation position (arbitrary) of the probe and (d) peak PA signal was plotted, p=0.0174. C-scan images of cuvettes from (c) containing labeled (e) C4-2 and (f) PC3 cells.

Quantitative characterization of this TMIA shows specific and uniform binding to PSMA-expressing PrCa cells. Four samples of ten million PC3 or C4-2 cells were labeled as above and imaged at 770 nm [Fig. 4(a)] and fluorescence emission was quantitated [Fig. 4(b)] using an IVIS spectrum (Perkin Elmer). TMIA labeling was uniform (CV=5.5% for PC3, 6.0% for C4-2) and the signal (C4-2) to noise (PC3) ratio (SNR) was 2.56±0.08. One of the four TMIA-labeled cell samples was used to tune the PA system alignment, and the remaining three were sequentially pipetted into the PA cuvette (Fig. 1) and peak PA signal determined at 785 nm excitation from a B-scan [Fig. 4(c)] for each of the six samples. When quantitated, the SNR was 2.33±0.09 [Fig. 4(d)]. The B-scan data were reconstructed as C-scan depicting each cuvette of C4-2 cells [Fig. 4(e)] and PC3 cells [Fig. 4(f)]. Analyzing the C-scan signal using ROIs encompassing each of the six cuvettes, the PA SNR was 5.35±0.04.

### 3.4 Quantitative Assessment of Inhibitor as Targeting Agent

**Fig. 5:**
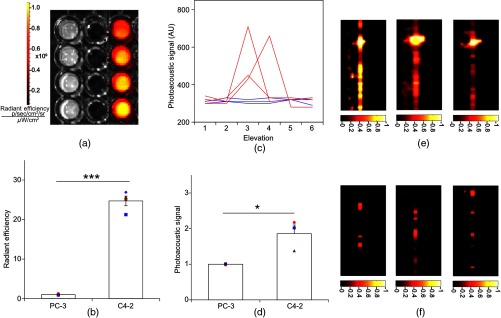
Enhanced photoacoustic signal from PSMA+ C4-2 PrCa cells labeled with YC27 TMIA. Optical imaging of a multiwell plate containing aliquots of PSMA+ C4-2 (right side four wells) and PSMA- PC3 (left side four wells) cells labeled with urea-dye (buffer in middle wells). Fluorescent signal intensity was captured via (a) IVIS and (b) relative intensity plotted, p<0.001. (c) Cells from three of each of the wells in (a) were sequentially loaded into the PAI instrument cuvette (Fig. 1) and PA signal (B-scan) was captured at 785 nm from c4-2 (red lines) and PC3 (blue lines), by scanning the elevation (long) axis of the cuvette, elevation position (arbitrary) of the probe and (d) peak PA signal was plotted, p=0.0246. C-scan images of cuvettes containing C4-2 (e) and PC3 (f).

An alternate targeting moiety, consisting of the DCL inhibitor conjugated to IRDye800CW (YC-27, Licor) was similarly evaluated as a TMIA. As above, there was a 20-nm shift in the wavelength of absorbance producing peak PA signal, to 785 nm (DNS). The YC-27 TMIA also shows specific and uniform binding to PSMA-expressing PrCa cells as assessed by fluorescence imaging at 770 nm [Figs. 5(a) and 5(b)]. Variability of YC-27 TMIA labeling was somewhat higher than for the aptamer TMIA (CV=17% for PC3, 9.9% for C4-2), but the fluorescent SNR was much larger, 24.7±1.22 (versus 2.56 for the aptamer). Peak PA signal from a B-scan for each cell type after labeling with the YC-27 TMIA [Fig. 5(c)] produced an SNR of 1.86±0.24 [Fig. 5(d)]. Figures 5(e) and 5(f) are C-scans depicting each cuvette of C4-2 cells and PC3 cells, respectively. Analyzing the C-scans using an ROI encompassing each cuvette, the PA SNR was 5.91±0.81.

## 4 Discussion

TMIAs optimized for PAI, in combination with an acoustic lens, allowed us to discriminate between PrCa cells lines that either express PSMA or do not express PSMA. Both the A10 aptamer and the DCL inhibitor specifically bind PSMA-expressing cells and are compatible i.p. or intratumoral delivery *in vivo*.^10^^,^^17^^,^^18^ While the aptamer TMIA showed slightly better SNR for PAI, it is expensive to synthesize and less stable than the inhibitor. The YC-27 is much brighter by fluorescence and conjugation of modified chromophores to the DCL inhibitor is more tractable.^18^ The urea-targeting agent therefore represents a better approach to developing “louder” TMIAs that will be necessary to overcome the challenges to detecting PrCa by PAI *in vivo*, intensity of light penetration to deep tissue, and TMIA-chromophore abundance due to target density from small tumors.
